# On the rise or a return to pre-pandemic levels? A cross-sectional online survey on nicotine, alcohol, and illicit drug use among youth

**DOI:** 10.1007/s40211-024-00503-5

**Published:** 2024-08-14

**Authors:** Selina Fanninger, Anna Mayer, Andreas Goreis, Oswald D. Kothgassner, Julia Matjazic, Paul Schoegl, Nicolas Schmelzle, Valentin Wollenek, Katrin Skala

**Affiliations:** 1 Department of Child and Adolescent Psychiatry, Medical University Vienna, Spitalgasse 23, 1090 Vienna, Austria; 2 Comprehensive Center for Pediatrics (CCP), Medical University of Vienna, Vienna, Austria

**Keywords:** COVID-19, Substance use, Youth, Nicotine, Alcohol, Cannabis, COVID-19, Substanzgebrauch, Jugend, Nikotin, Alkohol, Cannabis

## Abstract

**Objectives:**

The COVID-19 pandemic has had unprecedented and deteriorating effects on the mental health of adolescents and young adults. Various studies have described changes regarding substance abuse, but findings are conflicting.

**Study design:**

We conducted a cross-sectional online survey on nicotine, alcohol, and illicit drug use.

**Methods:**

From March to May 2023, 502 participants aged 14–24 from a community-based sample completed the questionnaire.

**Results:**

We found a general trend of declining or stable substance use during the first 2 years of the pandemic; however, in the third year (i.e., 2022), substance use returned to pre-pandemic levels or exceeded it. Compared with young adults (age 19–24), adolescents’ (age 14–18) use increased more clearly. Participants who scored above the cut-off on screening measures for problematic substance use showed a more pronounced increase in the use of cigarettes and illicit drugs but not of alcohol. Higher alcohol consumption during lockdowns was associated with increased likelihood of current problematic alcohol (odds ratio [OR]: 3.03) and cannabis use (OR: 2.60). Furthermore, individuals who reported increased usage of one psychotropic substance during lockdowns were more likely to have increased their use of other substances as well (OR: 2.66–4.87).

**Conclusions:**

Although not optimally generalizable due to the retrospective online format and convenience sampling, our results support the notion that special attention ought to be paid to certain subgroups such as younger people and those who already exhibit problematic substance use during the pandemic. Following up on post-pandemic trends in substance use is crucial for developing prevention measures and targeted interventions.

## Abstract (de)

**Ziel:**

Die COVID-19-Pandemie hatte nie dagewesene negative Auswirkungen auf die psychische Gesundheit von Jugendlichen und jungen Erwachsenen. In verschiedenen Studien wurden Veränderungen des Substanzmissbrauchs beschrieben, aber die Ergebnisse sind widersprüchlich.

**Studiendesign:**

Es wurde eine Online-Querschnittserhebung zum Konsum von Nikotin, Alkohol und illegalen Drogen bei Jugendlichen durchgeführt.

**Methode:**

Von März bis Mai 2023 füllten 502 Teilnehmende im Alter von 14–24 Jahren aus einer Bevölkerungsstichprobe den Fragebogen aus.

**Ergebnisse:**

Es stellte sich eine allgemeine Tendenz zu abnehmendem oder stabilem Substanzkonsum während der ersten 2 Jahre der Pandemie heraus, im dritten Jahr (d. h. 2022) kehrte der Substanzkonsum allerdings auf ein Niveau wie vor der Pandemie zurück oder überstieg es sogar. Im Vergleich zu jungen Erwachsenen (Alter: 19–24 Jahre) stieg der Konsum bei Jugendlichen deutlicher an (Alter: 14–18 Jahre). Teilnehmer mit Werten oberhalb des Grenzwerts bei Screening-Parametern für problematischen Substanzgebrauch wiesen einen deutlicheren Anstieg bei dem Konsum von Zigaretten und illegalen Drogen, nicht aber von Alkohol auf. Ein höherer Alkoholkonsum während der Lockdowns ging mit einer erhöhten Wahrscheinlichkeit für aktuellen problematischen Alkohol- (Odds Ratio [OR]: 3,03) und Cannabiskonsum (OR: 2,60) einher. Außerdem war es bei Personen, die einen erhöhten Konsum einer psychotropen Substanz während der Lockdowns angegeben hatten, wahrscheinlicher, dass sie ihren Konsum anderer Substanzen ebenfalls gesteigert hatten (OR: 2,66–4,87).

**Schlussfolgerung:**

Auch wenn die vorliegenden Ergebnisse nicht optimal zu verallgemeinern sind, da ein retrospektives Online-Format und eine Gelegenheitsstichprobe angewendet wurden, unterstützen sie die Auffassung, dass bestimmten Untergruppen besondere Aufmerksamkeit geschenkt werden sollte, wie jüngeren Leuten und Personen, die bereits während der Pandemie einen problematischen Substanzgebrauch aufgewiesen haben. Die laufende Beobachtung postpandemischer Tendenzen beim Substanzkonsum junger Menschen ist von entscheidender Bedeutung für die Entwicklung von Präventionsmaßnahmen und gezielten Interventionen.

## Introduction

Following the World Health Organization’s declaration of the novel coronavirus SARS-CoV 2 (COVID-19) as a pandemic in March 2020, numerous government-mandated restrictions (e.g., lockdowns, contact restriction, remote schooling) were implemented as preventive measures to curb infection rates [19]. These had unprecedented ramifications on day-to-day life and, subsequently, on the mental health of children and adolescents [10, 22, 23, 34]. Stay-at-home orders and the increased time spent at home instead of with peers seemingly disrupted youths’ development [17].

Several studies have shown changes in adolescent alcohol, nicotine, and illicit drug use since the start of the pandemic; however, findings have largely been inconclusive. Perceived social isolation during the pandemic has been described as being related to substance use in adults [4, 27]. A study reported that approximately one-fourth of respondents (24%) expressed feelings of isolation and uncertainty about where to find support, while almost half of the participating students (47%) claimed that their levels of stress and anxiety had been adversely impacted by the pandemic.

In a study by Romano et al. [24], 12% of students reported using cigarettes, alcohol, or cannabis specifically as a coping mechanism for COVID-19-related changes. Higher self-reported levels of stress predicted increased cigarette and e‑cigarette use during the pandemic in youth and young adults [6].

Even though the perceived availability of nicotine products, alcohol, and cannabis declined during the pandemic, self-reported prevalence did not [18]. For cannabis, however, findings were inconsistent [5, 6].

Substance use among adolescents and young adults is a major public health concern. Young people’s brains are still developing, making them more vulnerable to the negative effects of drug, alcohol, and nicotine use. The heightened reactivity to the release of dopamine within the mesolimbic system combined with an elevated likelihood of engaging in risky consumption behavior due to synaptic pruning makes them particularly vulnerable to addictive behaviors. The consumption of psychotropic substances has a disproportionately harmful impact on the physical well-being as well as on the development of adolescents compared with adults. Many behaviors—both adaptive and maladaptive (e.g., addictive behavior)—are established early in life. Research suggests that the age at which regular consumption is initiated is crucial to the severity of the consequences [25, 32].

While systematic reviews reported a tendency of declining or unchanged use of nicotine, e‑cigarettes, alcohol, and cannabis during the pandemic [15, 34], some studies have shown an increase in substance use, especially of “downers” (e.g., opiates, benzodiazepines), during the pandemic [29, 33]. The aim of the current study was to investigate whether and to what extent substance use has changed in the general population of youth.

## Method

An anonymous online survey was conducted from March to May 2023 using the platform SoSci Survey [16]. The survey included questions about recent and lifetime use of psychotropic substances, using questions from the 2018 HBSC (Health Behaviour in School-aged Children; [11]) and the 2019 ESPAD (European School Survey Project on Alcohol and Other Drugs; [13]) surveys. It further included questions pertaining retrospectively to substance use throughout the pandemic and screening measures for problematic substance use. Participants were required to provide informed consent prior to the start of the survey. There was no financial or other compensation for participation. The study procedure and questionnaire were approved by the Ethics Committee of the Medical University of Vienna (EK 1211/2023).

### Recruitment and participants

Participants were recruited from a community-based convenience sample in German-speaking countries (Austria, Germany, Switzerland). The study was promoted online on social media (e.g., Facebook, Instagram, Twitter, Reddit, WhatsApp) as well as via printed flyers and word of mouth. Participants had to be between 14 and 24 years.

### Measures

We collected the following demographic information: age, gender, current relationship status, country of residence, highest level of education completed, current occupation, and net monthly income.

Lifetime prevalence of nicotine, alcohol, cannabis, and other drugs was assessed. Past-year and past-month consumption of nicotine and nicotine products (cigarettes, water pipe/shishas, e‑cigarettes, and e‑shishas), alcohol in general and of specific alcoholic beverages (beer, wine, spirits, alcopops/mixed drinks, any other alcoholic beverage), and cannabis was assessed. On a bipolar 5‑point Likert scale (*significantly less*—*significantly more*), participants were asked to indicate whether their consumption of the respective substance had increased or decreased compared to 2 years ago and during lockdowns and compared to the most recent time before the lockdown. Furthermore, respondents were asked to retrospectively estimate on a continuous scale whether their nicotine, alcohol, and drug use had increased or decreased in quarter-yearly increments from spring 2020 to winter 2022/2023 compared to before the pandemic. Using questions from the ESPAD survey, motives for consuming alcohol were examined [13]. Participants were asked about the frequency of consuming five or more alcoholic beverages within a short period of time in the past month, which is the criterion for binge drinking [30]. They were also asked how many times in the last month they had been heavily drunk, meaning they were so intoxicated that their ability to speak or walk was impaired.

To screen for nicotine dependence, the Heaviness of Smoking Index (HSI; [12]) was employed. The self-report measure comprises two items: (1) the time interval between waking up and the first cigarette and (2) the number of cigarettes smoked per day. Cronbach’s α was 0.71 in the current sample. A composite score was calculated and 3 (indicating moderate addiction) was set as the cut-off for problematic nicotine use.

The CAGE (“Cut down drinking”, “Annoyed by criticism”, “Guilty feelings”, and “Eye-opener”; [7, 9]) questionnaire is a self-report measure designed to screen for alcohol dependence. It consists of four dichotomous (*yes*/*no*) items targeting signs of alcoholism (α = 0.70). A score of two or more indicates problematic alcohol use.

Lastly, the Cannabis Abuse Screening Tool (CAST; [28]) was employed. It includes six dichotomous (*yes*/*no*) items (α = 0.79), and a score above 3 indicates problematic cannabis use.

### Data analysis

We divided the sample into subgroups for analysis (by age: 14–18, 19–24, by gender: female, male, other). For the analyses, participants were also grouped into categories according to whether they scored above or below the clinical cutoff of the screening measures. A broader operationalization of problematic substance use was having scored above the cutoff on any of the screening tools. Participants who quit the survey before reaching the last page were excluded from analyses. Participants who did not complete individual items but reached the last page were not immediately excluded.

We conducted repeated-measures ANOVAs and calculated odds ratios. We aimed for at least 153 participants (assuming a small effect η^2^p = 0.1, power = 0.8). Data were analyzed using IBM SPSS statistics (version 28). The R package ggplot2 [31] was used to visualize data.

## Results

A total of 502 participants between the ages of 14 and 24 completed the survey, 60% were female and the average age was *M* = 19.96 years (*SD* = 2.63; Table 1). Of the respondents, 26.1% were high school students, 17.9% apprentices, 47.4% university students, 6% employees, 0.8% were unemployed, and 0.8% indicated that their current occupation was not listed among the options. Missing data were common and especially prevalent in the HSI questionnaire (*n* = 389) and CAST questionnaire (*n* = 399).

**Table 1:** Sample characteristics^a^

Variable	Age 14–18	Age 19–24	Total
	*n* = 152	*n* = 350	*n* = 502
*Age*	16.74 (1.24)	21.36 (1.66)	19.96 (2.63)
*Gender*			
Female	87 (57.2)	214 (61.1)	301 (60.0)
Male	61 (40.1)	132 (37.7)	193 (38.4)
Other	4 (2.6)	4 (1.1)	8 (1.6)
*Country*			
Austria	140 (92.1)	298 (85.1)	438 (87.3)
Germany	11 (7.2)	49 (14.0)	60 (12.0)
Switzerland	0 (0)	3 (0.9)	3 (0.6)
Other	1 (0.7)	0 (0)	1 (0.2)
*Current occupation*			
High school student	114 (75)	18 (5.1)	131 (26.1)
Apprentice	28 (18.4)	62 (17.7)	90 (17.9)
University student	8 (5.3)	230 (65.7)	238 (47.4)
Employee	1 (0.7)	29 (8.3)	30 (6.0)
Freelancer	0 (0)	1 (0.3)	1 (0.2)
Unemployed	0 (0)	4 (1.1)	4 (0.8)
Other	1 (0.7)	3 (0.9)	4 (0.8)

^a^*M* (SD); *n* (%)

### Prevalence

Among 14- to 24-year-olds, lifetime prevalence of cigarette use was 59%, while past-month prevalence was 34% (Table 2). Overall, 9% reported smoking cigarettes on a daily basis. With 50% and 52%, respectively, lifetime prevalences for e‑cigarettes and shisha were slightly lower than for cigarettes. Most participants (92%) had consumed alcohol at least once in their life. With 74%, the monthly prevalence was high as well. In total, 28% of respondents met screening criteria for problematic alcohol use. Lifetime prevalence for cannabis was 55%, while monthly prevalence was 25%. Overall, 27% of the participants who completed the CAST met criteria for problematic cannabis use. When comparing a subsample of youth aged 14–18 with data from the 2019 ESPAD study [13], we found that all prevalences in our sample were higher than in the ESPAD study: alcohol lifetime: 85% vs. 89%; alcohol past month: 60% vs. 71%; cigarettes lifetime: 48% vs. 57%; cigarettes past month: 25% vs. 37%; cannabis lifetime: 20% vs. 43%; and cannabis past month: 11% vs. 18%. Lifetime prevalence of many illicit drugs was higher as well. Compared with recent epidemiologic studies, three times as many participants reported ever having taken cocaine or amphetamines [14].

**Table 2:** Prevalence of (problematic) substance use^a^

Variable	Age 14–18	Age 19–24	Total
**Nicotine**			
*Cigarettes*			
Lifetime prevalence	86/151 (57.0)	206/342 (60.2)	292/493 (59.2)
Past 30 days	55/149 (36.9)	105/343 (30.6)	160/492 (33.9)
Daily smoking	14/152 (9.2)	33/349 (9.5)	47/501 (9.4)
HSI above cutoff	7/144 (0.5)	23/346 (0.7)	30/389 (0.8)
*E‑cigarettes, e‑shisha*			
Lifetime prevalence	88/151 (58.2)	159/345 (46.1)	247/496 (49.8)
Past 30 days	51/146 (34.9)	50/341(14.7)	101/487 (20.8)
*Hookah/shisha*			
Lifetime prevalence	57/152 (37.5)	200/347 (57.7)	257/499 (51.5)
Past 30 days	13/147 (8.8)	26/343 (7.6)	39/490 (8.0)
**Alcohol**			
Lifetime prevalence	131/148 (88.5)	316/341 (92.7)	447/489 (91.6)
Past 30 days	106/149 (71.1)	248/338 (63.9)	354/487 (73.5)
CAGE above cutoff	46/138 (30.3)	92/329 (28.0)	138/467 (27.5)
**Cannabis**			
Lifetime prevalence	65/150 (43.3)	203/346 (58.7)	268/496 (54.5)
Past 30 days	26/144 (18.1)	79/340 (23.2)	105/484 (24.5)
CAST above cutoff	19/118 (12.5)	87/281 (31.0)	106/399 (26.6)
**Other substances (lifetime prevalence)**			
Heroin	1/152 (0.7)	6/350 (1.7)	7/502 (1.4)
Methadone	1/152 (0.7)	5/350 (1.4)	6/502 (1.2)
Cocaine	11/152 (7.2)	55/350 (15.7)	66/502 (13.1)
Psilocybin	1/152 (0.7)	31/350 (8.9)	32/502 (6.4)
Mescaline	1/152(0.7)	5/350 (1.4)	6/502 (1.2)
LSD	3/152 (2.0)	38/350 (10.9)	41/502 (8.2)
Amphetamines	5/152(3.3)	52/350 (14.9)	57/502 (11.4)
New psychoactive substances	2/152(1.3)	17/350 (4.9)	19/502 (3.8)
Prescription sleeping pills or tranquilizers (without prescription)	13/152 (8.6)	36/350 (10.3)	49/502 (9.8)
Prescription sleeping pills or tranquilizers (with prescription)	16/152 (10.5)	50/350 (14.3)	66/502 (13.1)
Prescription stimulants (without prescription)	4/152 (2.6)	21/350 (6)	25/502 (5.0)
Prescription stimulants (with prescription)	5/152 (3.3)	16/350 (4.6)	21/502 (4.2)
Other	9/152 (0.6)	24/350 (6.9)	33/502 (6.6)

*CAGE* Questionnaire for Alcohol Misuse, *CAST* Cannabis Abuse Screening Tool, *HSI* Heaviness of Smoking Index

*n* (%)

In a cross-sectional study among Austrian apprentices conducted in spring 2021 [20], 12.8% of participants scored above the cutoff for problematic alcohol use in the CAGE questionnaire. With 27.5% in the current sample, problematic alcohol use was more than twice as common (Table 2).

**Fig. 1:**
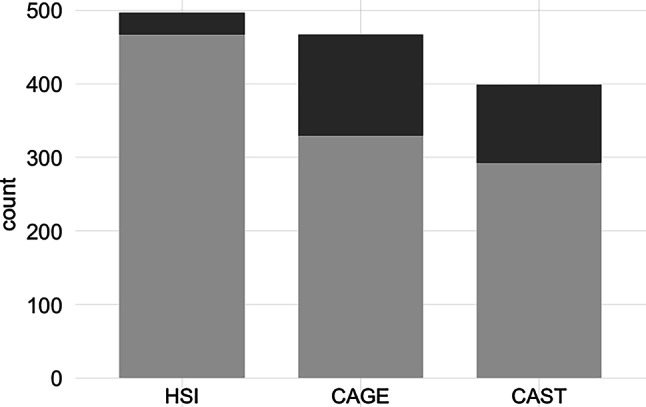
Participants who scored below the cutoff are represented in light gray; those who met cutoff criteria for problematic substance use are represented in dark gray. *CAGE* Questionnaire for Alcohol Misuse, *CAST* Cannabis Abuse Screening Tool, *HSI* Heaviness of Smoking Index

### Reasons for alcohol consumption

Participants could select various reasons for drinking alcohol. The most prevalent reasons were that it makes social gatherings more entertaining (47%), that it is fun (43%), and that it helps with having fun at parties (37%). Over one-third enjoy the feeling of being drunk (37%). Other reasons included coping, such as cheering oneself up when one is in a bad mood (18%), forgetting problems (12%), or helping with feelings of depression or nervousness (12%). Almost 10% stated that social pressure, fear of being left out (7%), and to be more likable (3%) led them to consume alcoholic beverages.

### The impact of COVID

**Fig. 2:**
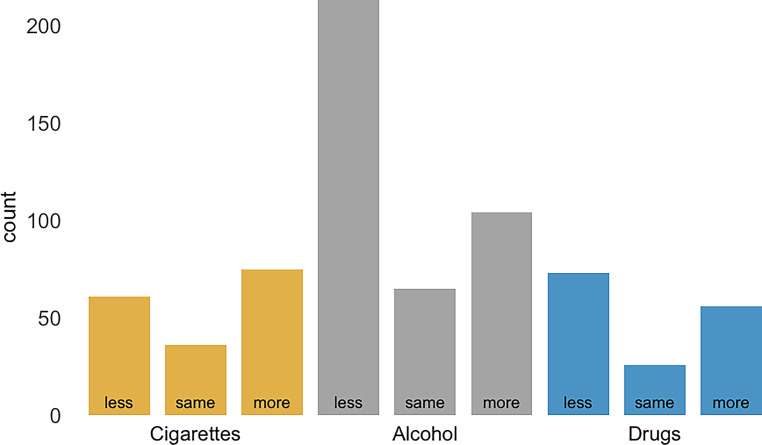
Change in substance use during lockdowns, reported retrospectively

In line with previous research, the use of alcohol and illicit drugs largely declined during lockdowns. However, a substantial number of participants (*n* = 75, 44%) reported smoking more cigarettes, compared with 35% (*n* = 61) who reported smoking fewer cigarettes, and 21% (*n* = 36) who did not report a change in their frequency of smoking. Alcohol consumption decreased in 56% (*n* = 213) of participants, while 17% (*n* = 65) reported no change, and 27% (*n* = 104) reported drinking more alcohol during lockdowns. Among participants who used illicit drugs, almost half (47%; *n* = 73) reported using less, 17% (*n* = 26) reported having used the same amount, and 37% (*n* = 56) increased their drug use during lockdowns (see Fig. 2).

Most participants stated not having been heavily drunk in the past month (86%). Half of the participants reported not having drunk more than five alcoholic beverages on one occasion, 16% reported having done so once in the past month, 14% twice, 13% 3–5 times, 3% 6–9 times, and 2% 10 or more times.

To assess the effect of time (quarter-yearly increments from spring 2020 to winter 2022/2023) on the frequency of nicotine, alcohol, and illicit drug consumption, repeated-measures ANOVAs with Greenhouse–Geisser corrections were performed. There were statistically significant effects of time on the frequency of alcohol consumption between at least two time points on the use of nicotine, *F*(2.99,583.59) = 20.03, *p* < 0.001, alcohol, *F*(4.1,1309.2) = 40.54, *p* < 0.001, and illicit drugs, *F*(3.72,566.11) = 15.18, *p* < 0.001. Post hoc tests revealed that only the use of alcohol changed significantly at two consecutive points—from spring 2020 to summer 2020 (*p* < 0.001, *d* = 0.15) and from spring 2021 to summer 2021 (*p* < 0.001, *d* = 0.20)—while there was no significant change of nicotine and illicit drug use when assessing consecutive time points.

Between-subjects Greenhouse–Geisser corrected repeated-measure ANOVAs were performed to assess whether adolescents and young adults with problematic substance use (i.e., scoring above the cutoff for any of the employed screening questionnaires) increased their substance use during the pandemic, compared with youth not reporting problematic substance use. There was a statistically significant difference in frequency of use between at least two time points between the groups for alcohol, *F*(4.09, 1300.28) = 2.46, *p* = 0.04, but not for nicotine, *F*(3.02, 585.98) = 2.47, *p* = 0.06, and illicit drugs, *F*(3.77, 568.87) = 2.06, *p* = 0.09.

**Fig. 3:**
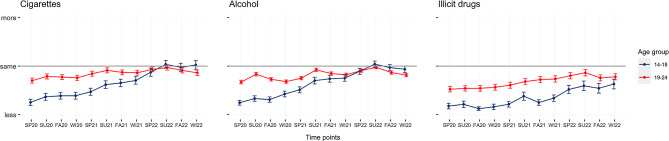
Trends in the frequency of substance use during the pandemic, by age group, for three different substance classes. *SP* spring, *SU* summer, *FA* fall, *WI* winter

**Fig. 4:**
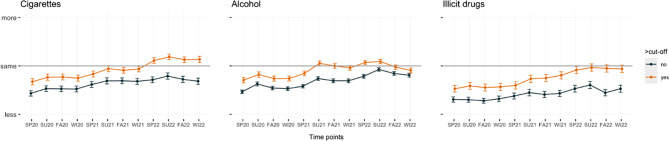
Trends in the frequency of substance use during the pandemic, grouped by score on screening measures, for three different substance classes. *SP* spring, *SU* summer, *FA* fall, *WI* winter

Participants who scored above the cutoff on screening measures for problematic substance use reported a higher frequency of substance use at all time points, compared with participants scoring below the cutoff (Figs. 3, 4).

Participants who reported smoking more cigarettes or drinking more alcohol during lockdowns were not more likely to currently show problematic nicotine use (odds ratio [OR] 1.74, 95% confidence interval [CI]: 0.71–4.25; OR 0.95, 95% CI: 0.37–2.45).

Those who drank more alcohol during lockdowns were more likely to currently exhibit problematic alcohol (OR 3.03, 95% CI: 1.90–4.85) and cannabis (OR 2.60, 95% CI: 1.56–4.33) use.

Individuals who reported using more of one specific substance during lockdowns were more likely to have increased their use of other substances as well (alcohol and cigarettes OR = 2.66, 95% CI: 1.37–5.15; alcohol and illicit drugs OR = 4.87, 95% CI: 2.36–10.04; cigarettes and illicit drugs OR = 3.48, 95% CI: 1.56–7.78).

## Discussion

The objective of the current study was to assess the current and past prevalence of substance use and to retrospectively explore changes in substance use patterns in adolescents and young adults in the course of the COVID-19 pandemic.

In line with previous research [3, 15, 34], our results show declined use in a community-based sample compared with before the pandemic. However, over time, there was a trend of returning to pre-pandemic levels. Post hoc analyses indicated that there only were significant changes in alcohol consumption at two consecutive time points: from spring 2020 to summer 2020 (*p* < 0.001, *d* = 0.15) and from spring 2021 to summer 2021 (*p* < 0.001, *d* = 0.20). However, there were no changes in nicotine and illicit drug use when evaluating consecutive time periods. One reason for the increase in alcohol consumption during summer might be its prevalence at social events. Almost half of the participants stated that alcohol makes social gatherings more entertaining. Due to school holidays, vacations, and the relaxation of lockdown measures there may have been an increased number of opportunities for such events. These results are in line with Benschop et al. [2], who identified having fewer social occasions than “pre-corona” as the most important reason for decreased substance use.

Our findings suggest that the COVID-19 pandemic did not lead to an overall increase in the prevalence of substance use. However, some subgroups might have increased their use during this period. Youth who scored above the cutoff on screening measures increased their substance use more significantly during the pandemic than those who scored below the cutoff. These participants reported a higher frequency of substance use than participants who scored below the cutoff at all time points (Fig. 4). A clinically significant difference between the consumption frequency of the two groups was identified only for alcohol, *F*(4.09, 1300.28) = 2.46, *p* = 0.04, but not for nicotine, *F*(3.02, 585.98) = 2.47, *p* = 0.06, and illicit drugs, *F*(3.77, 568.87) = 2.06, *p* = 0.09.

Despite an overall decline in consumption during the analysis period, adolescents exhibiting conspicuous consumption patterns did not experience a comparable decrease. Youths who scored above the designated cutoff on screening measures showed a greater increase in substance use during the pandemic compared with those who scored below the cutoff. While the subgroup who scored above the cutoff consistently demonstrated a higher frequency of substance use at all time points, only the differences in alcohol consumption were statistically significant, *F*(4.09, 1300.28) = 2.46, *p* = 0.04. Both nicotine, *F*(3.02, 585.98) = 2.47, *p* = 0.06, and illicit drugs, *F*(3.77, 568.87) = 2.06, *p* = 0.09, did not show statistical significance. The data do not provide sufficient evidence to draw conclusions about the causes of this consumption behavior. However, previous studies support the hypothesis that the COVID-19 pandemic measures might have disproportionately impacted certain subgroups. This specific increase in at-risk groups is attributed to familial issues [21], pandemic-related stress [5], socioeconomic challenges [21], and social isolation [3]. Additionally, the pandemic might have contributed to an increase in solitary consumption [8], which is linked to factors such as increased alcohol consumption, social discomfort, and negative affect [26].

Furthermore, the data suggest differences in usage patterns between the two age categories (14–18 and 19–24). Throughout most of the observed assessments, the cohort comprising young adults demonstrated a higher frequency of consumption compared to their adolescent counterparts. Notably, there was a gradual decrease over time in the discrepancy in consumption rates between the two groups. Although there was a notable disparity in consumption patterns during the summer of 2020, adolescents and young adults showed a more similar profile for cigarettes and alcohol during the summer of 2022.

### Limitations

Due to the use of a cross-sectional design in the present study and the focus on prevalence, it is important to note that no causal conclusions can be drawn from our findings. We asked participants to self-report their substance use retrospectively, and this could introduce recall bias resulting in either under- or overestimating the actual extent of change. Participants might also either idealize their pre-pandemic consumption behavior or underestimate the current extent of substance use.

Because of convenience and snowball sampling, as well as self-selection and under-coverage inherent to online surveys [1], our findings are not optimally generalizable to the general young population. Our sample overrepresents youth who use substances, university students, and females. The proportion of diverse individuals is too small to conclude on robust results from this subgroup. Moreover, due to the anonymity of participants, we cannot exclude the possibility of duplicate responses. However, we consider this probability to be low, as no incentives were provided. The current study was not intended to be epidemiological but instead aimed to explore changes in substance use patterns among German-speaking youth. Thus, the actual prevalence of problematic substance use may be lower. We did not ask for pandemic-specific reasons, and thus no conclusions can be drawn regarding the causes for the changes in substance use or the purposes attributed to it.

## Conclusion

In this study, we conducted a cross-sectional online survey to investigate changes in nicotine, alcohol, and illicit drug use among youth during the COVID-19 pandemic. Our findings indicated a trend of declining or stable substance use during the initial years of the pandemic, followed by a return to pre-pandemic levels or even an increase in the third year. Adolescents exhibited a more pronounced increase in substance use compared with young adults. Those who scored above the cutoff for problematic substance use on screening measures showed a greater increase in cigarette and illicit drug, but not alcohol use. Increased alcohol consumption during lockdowns was associated with a higher likelihood of problematic alcohol and cannabis use. Moreover, participants who reported increased usage of one psychotropic substance during lockdowns were more likely to have increased their use of other substances as well. While the pandemic seemed to result in initial decreases in substance use, especially during lockdown periods, the subsequent return to pre-pandemic levels or even higher use suggests that the impact of the pandemic on youth substance use is complex and multifaceted. These findings underscore the importance of continued monitoring of substance use trends among young people and the need for targeted interventions for specific subgroups, particularly those with pre-existing problematic substance use or mental health issues.

It is crucial for policymakers, educators, and healthcare professionals to recognize the potential impact of prolonged periods of isolation, stress, and disrupted routines on youth substance use behaviors. Prevention strategies and interventions should consider the dynamic nature of substance use patterns and the interplay between substance use, mental health, and social factors. As the world navigates the post-pandemic phase, these insights can inform the development of effective measures to mitigate the potential negative consequences of substance use among adolescents and young adults. Additionally, further research using longitudinal designs and face-to-face interviews will be valuable in obtaining a more comprehensive understanding of the complex relationship between the pandemic and youth substance use.

## Data Availability

The data that support the findings of this study are available from the corresponding author, KS, upon reasonable request.
